# Efficacy of Raman spectroscopy in the diagnosis of hepatocellular carcinoma: A protocol for systematic review and meta-analysis

**DOI:** 10.1097/MD.0000000000023884

**Published:** 2020-12-24

**Authors:** Hongyu Jin, Man Zhang, Kaiyu Jia, Libin Huang

**Affiliations:** a Department of Liver Surgery & Liver Transplantation Center, West China Hospital, Sichuan University; b Department of Gynecology and Obstetrics, West China Second University Hospital, Sichuan University; Key Laboratory of Obstetric & Gynecologic and Pediatric Disease and Birth Defects of Ministry of Education; c West China School of Medicine; d Department of Gastroenterology, West China Hospital, Sichuan University, Chengdu, China.

**Keywords:** Raman spectroscopy, hepatocellular carcinoma, sensitivity, specificity, accuracy

## Abstract

**Background:**

To comprehensively analyze the relative effectiveness of Raman spectroscopy (RS) in the diagnosis of suspected hepatocellular carcinoma (HCC).

**Methods:**

We will perform a complete systematic review based on studies from PubMed/Medline, EMBASE, Web of Science, Ovid, Web of Knowledge, Cochrane Library and CNKI. We plan to identify over 2000 spectra with strict criteria in several individual studies published between January 2008 and November 2020 in accordance to Preferred Reporting Items for Systematic Reviews and Meta-Analysis (PRISMA) guidelines. We are going to summarize the test performance using random effects models.

**Results:**

This study will provide solid evidence and data on the sensitivity, specificity and accuracy of RS in the diagnosis of HCC.

**Conclusion:**

Through this meta-analysis, we intend to provide the pooled sensitivity, specificity and diagnostic accuracy of RS in the diagnosis of suspected HCC. Other parameters like positive LR, negative LR, DOR and AUC of the SROC curve will also be calculated and drawn to help illustrate the efficacy of RS in the diagnosis of HCC.

## 1 Introduction

Hepatocellular carcinoma (HCC) is one of the severest malignancies in the world characterized by a comparatively high prevalence and disease-related burden due to high recurrence rate and inability to full fill early diagnosis. With respect to cancer incidences, primary liver cancer ranked number 7 among all cancer catagories and even higher in abdominal cancer. Meanwhile, it ranked number 4 in cancer-related mortality worldwide in 2019 alone. Among all histological subtypes of primary liver cancer, HCC is regarded as the most common and relatively severe type, which accounts for about 75% of all cases reported. In recent years, despite the decreasing morbidity of old contributing factors like hepatitis B virus (HBV) infection and abnormally high alcohol intake, novel emerging factors are continuously being discovered. Thus, despite the constantly fluctuating epidemiology, the influence and disease burden of HCC are hardly cut down.^[1]^

Currently, the most effective and eventually curative treatment of HCC is regarded as radical surgery after the determination of malignance either pathologically or by image, which directly resects lesions and suspected areas so that patients are supposed to have a satisfactory prognosis.^[2]^ However, pre-surgical liver puncture is known to have several shortcomings, such as the possibility of bleeding and tumor metastasis.^[3]^ Therefore, liver puncture is not regularly done before surgery, while most of liver masses tend to be determined through image. Nevertheless, sometimes it can be difficult to judge the benign and malignant essence of the liver masses discovered.

In recent years, Raman spectroscopy (RS) has been introduced into clinical practice for their capacity to tell apart the benign and malignant essence of tumor. Moreover, RS is also applied during surgery to aid surgeons by identifying the exact borderline between malignant and normal tissue since it can optically characterize the internal compositional properties.^[4,5]^ Meanwhile, RS examination can be carried out in vivo and compared with traditional imaging technologies, it is real-time, label-free and nondestructive.^[6,7]^ According to its theory, RS detects variation of wave-lengths or Raman shifts induced by inelastic light scattering from certain molecules.^[8]^ Because molecules have distinct combinations of Raman shifts, molecules of different property proposition are supposed to produce unique spectral signatures.^[9]^ Therefore, measured Raman spectra can provide evidence of the internal compositional features of tissues. In additionally, the availability to be examined in vivo and its label-free, real-time and non-destructive characteristics perfectly addresses the deficiencies of traditional liver puncture.

In the past decade, a number of clinical researches trying to confirm the diagnostic accuracy, sensitivity and specificity of RS in the diagnosis of HCC have been widely launched and several significant, meaningful outcomes have been generated. Thus, in order to comprehensively analyze the exact diagnostic efficiency of RS in determining the benign and malignant features of HCC, we plan to carry out this meta-analysis and systematic review in order to define the clinical value of RS.

## 2 Material and methods

This protocol has been registered on the International Platform of Registered Systematic Review and Meta-analysis Protocols (INPLASY registration number: INPLASY2020110089; INPLASY DOI number: 10.37766/inplasy2020.11.0089. Available at: https://inplasy.com). We will document essential protocol amendments in the full review and update information in the registry. This study has been approved by the Ethics Committee of West China Hospital, Sichuan University (Chengdu, China).

### 2.1 Search strategy

Relying on the guidelines for performing meta-analysis, we will search extensively acknowledged authenticated databases including PubMed/Medline, Web of Science, Cochrane Library, ClinicalTirals.gov (http://www.ClinicalTrials.gov), China National Knowledge Infrastructure (CNKI) for related articles published from January 2008 to November 2020. Articles we primarily searched and identified will be subsequently screened for their quality, relevancy and availability. No language restriction will be used. The keywords (query) of our primary search will be as follows: (((((((Hepatocellular carcinoma) OR (HCC)) OR (liver tumor)) OR (liver mass)) OR (hepatocellular mass)) AND (Raman)) OR (Raman spectroscopy)) OR (RS)

### 2.2 Article selection

Two independent reviewers are planned to participate in the screening process to analyze the full texts and to perform quality assessments and relevancy determination. The main inclusion criteria will contain:

1. reporting the use of RS in hepatocellular carcinoma;
2. being a randomized controlled trial and/or using any observational designs, including cross-sectional, case-control and cohort designs;
3. reporting the sensitivity, specificity values or true positive (TP), false positive (FP), true negative (TN), and false negative (FN) values, based on which sensitivity and specificity values could be calculated.

Meanwhile, we particularly intend to exclude studies which are letters, editorials, case reports, etc. Subsequently, we will perform a blinded cross-check to detect underlying discrepancies. If a potential discrepancy is detected, a blinded third reviewer will be assigned to adjudicate the conflict. The identification, inclusion and exclusion of studies are going to be performed according to PRISMA guidelines.

### 2.3 Data extraction

Two experienced investigators plan to independently analyze the final defined articles for primary parameters which indicate the diagnostic efficiency and secondary parameters concerning the basic information of the article. During the process, unexpected discrepancies are planned to be carefully discussed and resolved. In general, a total of 9 important diagnostic efficiency related parameters will be extracted, including diagnostic sensitivity, specificity, accuracy, TP, TN, FP, FN values as well as spectra values. In addition, secondary parameters which reflect the baseline characteristics of the articles including title, first author, nationality, department, ethnicity, study design, sex and median age of the patients and enrollment year will also carefully be extracted.

### 2.4 Statistical analysis

Data will be extracted on either an article or study level when possible to reconstruct a 2 × 2 table, which we depend on to calculate sensitivity, specificity, positive predictive value (PPV), negative predictive value (NPV), odds ratios (ORs) and diagnostic likelihood ratios (DLRs) with their 95% confidence intervals (CIs). The forest plots will be generated to display sensitivity and specificity estimates using Meta-Disc version 1.4 (Clinical Biostatistics Unit, UK). To summarize test performance, 2 methods for meta-analysis diagnostic accuracy test will be used: the bivariate model and the hierarchical summary receiver operating characteristic (HSROC) model.^[10,11]^ We choose to use these methods to respect the binomial structure of diagnostic accuracy data, thus jointly summarizing paired measures simultaneously, e.g., sensitivity and specificity or, positive and negative likelihood ratios (LRs). Meanwhile, as a random effects approach, the bivariate/HSROC meta-analysis allow pooling results in view of knowing that heterogeneity is commonplace across included studies due to different or implicit thresholds. The said approach will be carried out by metandi (Meta-analysis of diagnostic accuracy using hierarchical logistic regression) command in STATA 14.2 (Stata Corp, USA).

Additionally, summary receiver operator characteristics (SROC) curves will be generated to assess the relationship between sensitivity and specificity. Meanwhile, the area under curve (AUC) will be simultaneously calculated to evaluate the overall performance of RS. An excellent diagnostic effect is defined when AUC value is between 0.9 and 1; good when AUC value is between 0.8 and 0.9; fair when AUC value is between 0.7 and 0.8; poor when AUC value is between 0.6 and 0.7. The diagnostic method is regarded as failure when AUC is between 0.5 and 0.6.^[12]^ The SROC curved is made through Meta-Disc version 1.4 (Clinical Biostatistics Unit, UK).

### 2.5 Quality assessment

Standard quality evaluation of the included studies will be performed based on the Quadas-2 tool.^[13]^ Particularly, the risk of bias will be obtained by RevMan 5.3 (The Cochrane Collaboration). The articles will be evaluated in the following processes: sequence generation (selection bias), allocation concealment (selection bias), blinding of participants and personnel (performance bias), blinding of outcome assessment (detection bias), incomplete outcome data (attrition bias), selective reporting (reporting bias) and others.

### 2.6 Publication bias

Publication bias will be evaluated through Deeks Funnel Plot Asymmetry Test (consider the existence of publication bias when *P* < .05). The Deeks Funnel Plot Asymmetry Test will be conducted by Stata 14.2 (StataCorp, USA)

## 3 Discussion

This systematic review and meta-analysis will evaluate the diagnostic efficiency of RS in HCC patients. Currently, traditional imaging techniques like ultrasound, CT and MRI as well as a pre-surgical pathological biopsy are regarded as main methods to help illustrate the essence of liver mass before a radical surgery. However, pathological biopsy tends to cause uncontrollable bleeding and unavoidable tumor metastasis, radiological methods sometimes have difficulty in judging the benign and malignant essence of tumor, RS has gained increasing popularity. In other solid tumors, like esophageal cancer, kidney cancer, bladder cancer etc, RS has been proved to have comparatively satisfactory diagnostic efficiency, manifested by high sensitivity, specificity and accuracy.^[14]^ Thus, through this systematic review and meta-analysis, we aim to reflect on the appliance of RS in liver mass.

## Author contributions

**Conceptualization:** Hongyu Jin, Man Zhang, Libin Huang.

**Data analysis:** Man Zhang.

**Data collection or management:** Hongyu Jin, Man Zhang.

**Data curation:** Hongyu Jin, Man Zhang.

**Formal analysis:** Hongyu Jin, Man Zhang.

**Funding acquisition:** Libin Huang.

**Investigation:** Hongyu Jin, Man Zhang, Kaiyu Jia.

**Methodology:** Hongyu Jin, Man Zhang, Kaiyu Jia.

**Project administration:** Man Zhang.

**Protocol/Project development:** Hongyu Jin, Man Zhang, Kaiyu Jia

**Resources:** Hongyu Jin, Libin Huang.

**Software:** Hongyu Jin, Man Zhang.

**Visualization:** Libin Huang.

**Writing – original draft:** Hongyu Jin, Man Zhang, Libin Huang.

**Writing – review & editing:** Hongyu Jin, Libin Huang.

## Correction

When originally published, Dr. Hongyu Jin's name was spelled incorrectly as Hong Yu Jin and has since been corrected.
